# Venous Thrombosis Has a Constellation of Different Risk Factors: A Case Report and State-of-the-Art Review

**DOI:** 10.7759/cureus.30766

**Published:** 2022-10-27

**Authors:** Margarida Silva Cruz, Ligia Rodrigues Santos, Tiago Esteves Rodrigues, Francisco Manuel Pereira da Silva, Vera Ferraz Moreira

**Affiliations:** 1 Internal Medicine, Centro Hospitalar do Tâmega e Sousa, Penafiel, PRT; 2 Radiology, Centro Hospitalar do Tâmega e Sousa, Penafiel, PRT

**Keywords:** thrombophilia screen, mthfr a1298c mutation, hyperhomocysteinemia, pylephlebitis, portal vein thrombosis

## Abstract

Splanchnic venous thrombosis and cerebral venous thrombosis are uncommon manifestations of venous thromboembolism (VTE) that have been associated with inherited thrombophilias. Hyperhomocysteinemia is an established risk factor for thrombosis, and methylenetetrahydrofolate reductase (MTHFR) mutation is the most common genetic alteration in this condition. The association between MTHFR mutations, mild to moderate elevations in homocysteine, and the risk for thrombosis is controversial. Pylephlebitis, also known as suppurative portal vein thrombophlebitis, usually originates from an intra-abdominal infectious process. It is a condition with high morbidity and mortality, partly due to its late diagnosis, and antibiotics are the gold standard treatment. The purpose of anticoagulation is dubious. We describe the case of a 60-year-old male with a previous history of venous sinus thrombosis and MTHFR A1298C mutation with mild homocysteine ​​elevation who presented with signs and symptoms of intra-abdominal infection and whose abdominopelvic computed tomography (CT) with intravenous contrast showed splanchnic-vein thrombosis. Through this complex case, the authors present a review of the current state of the art on VTE, hyperhomocysteinemia, and pylephlebitis, emphasizing the need for a holistic view of the patient in the decision-making process.

## Introduction

Venous thromboembolism (VTE) is a thrombotic event that occurs in the venous circulatory system and whose pathogenesis is thought to result from alterations in blood flow (i.e., stasis) in the constituents of the blood (i.e., inherited or acquired hypercoagulable state) and vascular endothelial injury, also known as Virchow's triad [1,2]. Its estimated age- and sex-adjusted annual incidence rate ranged from 1.2-1.4 per 1,000 person-years, and its 30-day mortality rate could be as high as 10% [2]. VTE most often manifests as deep vein thrombosis in the lower limbs and pulmonary thromboembolism [2]. Cerebral venous and splanchnic-vein (portal, hepatic, splenic, or mesenteric) thrombosis represent unusual forms of VTE [3]. Inherited thrombophilias have been described to be correlated with a higher risk of VTE in these uncommon areas [3].

Hereditary risk factors such as mutation of proteins C, and S, antithrombin deficiency, factor V Leiden mutation, and prothrombin G20210A mutation or acquired, such as neoplasms, antiphospholipid syndrome, oral contraceptives, obesity, trauma, surgery, primarily orthopedic, among others, are associated with increased risk of venous thrombosis [2,3]. There is often a constellation of different risk factors in patients with a venous thrombotic event [2,3].

## Case presentation

**Figure 1:**
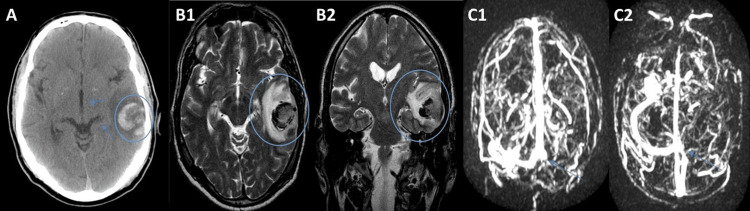
Left lateral venous sinus thrombosis (A) Cranioencephalic computed tomography (CT) shows a heterogeneous left temporal cortico-subcortical hemorrhagic lesion (circle) with a slight mass effect on the temporal horn and slight deviation of midline structures (arrows) but no brain hernias. (B) Brain magnetic resonance (MR) shows heterogeneous left temporal cortico-subcortical hematoma with surrounding edema (circle) that causes effacement of the adjacent sulci and slight deformation of the lateral ventricle - (B1) axial plane; (B2) coronal plane. (C) Brain MR angiography shows signal alteration of the lateral venous sinus on the same side with no flow in the venous angiogram obtained, suggestive of venous thrombosis (arrows) - (C1) coronal plane; (C2) axial plane.

A 60-year-old Caucasian male presented to the emergency department with a chief complaint of abdominal pain in the epigastric region and right hypochondrium with dorsal irradiation, chills, and a fever, for five days. His past medical history included hypertension, dyslipidemia, diverticulosis, and, five years ago, left lateral venous sinus thrombosis (Figures 1A-1C).

The etiological study revealed homozygosity for the methylenetetrahydrofolate reductase (MTHFR) A1298C mutation and mild homocysteine ​​elevation without other changes (Table 1).

**Table 1:** Thrombophilia screening tests *The following mutations were screened by microarray hybridization: c.1691G>A (p.R506Q) in the factor V gene (F5), g.20210G>A in the factor II gene (F2), c677C>T (p.A222V) and c.1298A>C (p.E429A) in the MHTFR gene. ^+^The c.1298C allele in homozygosity is associated with a decrease in methylenetetrahydrofolate reductase enzyme activity and hyperhomocysteinemia. Hyperhomocysteinemia represents a 1.5-fold increased risk of venous thrombosis, particularly if low folate levels.

Laboratory findings	Patient values	Reference range
Protein S deficiency (%)	102,8	63,5-149
Protein C deficiency (%)	95	71,8-146,2
Anti-thrombin-III deficiency (%)	75	83-128
Factor VII (%)	151	50-129
Homocysteinemia (µmol/L)	15,54	5-15
Lupus anticoagulante	Negative	-
Cardiolipin antibodies	Negative	-
B2-glycoprotein-1 antibodies	Negative	-
Prothrombin G20210A mutation*	Negative	-
Factor V Leiden R506Q mutation*	Negative	-
MTHFR C677T mutation*	Negative	-
MTHFR A1298C mutation*^+^	Homozygosity	-

Relevant family history was excluded. He completed 12 months of anticoagulation and began with folic acid and acetylsalicylic acid. In addition, his home medications included an angiotensin-converting enzyme (ACE) inhibitor, a thiazide diuretic, and fenofibrate-statin combination therapy.

**Figure 2:**
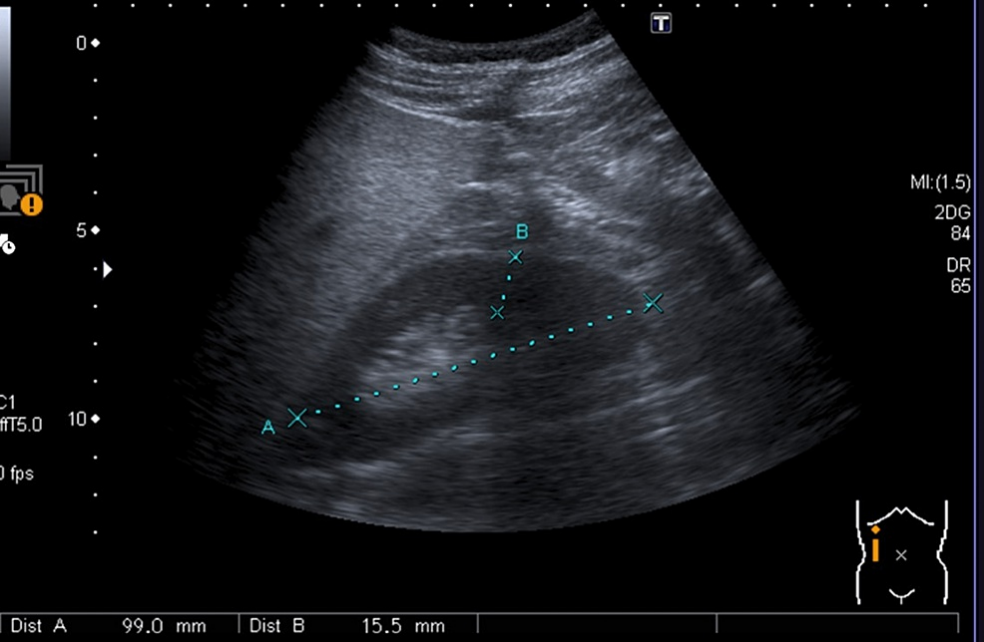
Abdominal ultrasound Kidney and right lobe of the liver with apparently standard dimensions.

On presentation in the emergency department, the patient was febrile and had sinus tachycardia. His examination was regular, except for mild tenderness on deep palpation of the epigastric region without guarding or rebound tenderness. The spleen and liver were non-palpable. Previous abdominal ultrasound showed no significant changes (Figure 2).

Bloodwork revealed mild thrombocytopenia, the elevation of C-reactive protein (CRP), slight hyperbilirubinemia with small increased liver cytolysis markers, gamma-glutamyl transferase (GGT), and creatine kinase (CPK) (Table 2).

**Table 2:** Baseline laboratory tests ALP, alkaline phosphatase; ALT, alanine transaminase; AST, aspartate transaminase; CPK, creatine kinase; CRP, C-reactive protein; GGT, gamma-glutamyl transpeptidase; WBC, white blood cells;

Laboratory findings	Patient values	Reference range
Hemoglobin (g/dL)	14,5	12-15
WBC (counts/µL)	9,870	4,500-11,000
Platelet (counts/µL)	143,000	150,000-400,000
CRP (mg/L)	351,2	<5
Creatinine (mg/dL)	1,1	< 1,3
Total bilirubin (mg/dL)	1,3	<1
ALT (U/L)	48	<31
AST (U/L)	47	<31
ALP (U/L)	60	34-104
GGT (U/L)	62	<49
Amylase (U/L)	32	28-100
Lipase (U/L)	31	22-51
CPK (U/L)	397	<172

**Figure 3:**
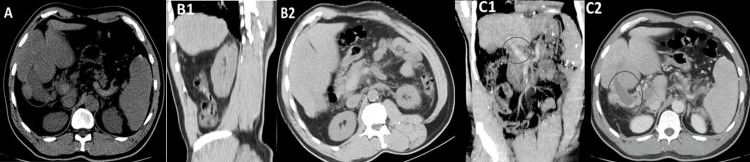
Abdominopelvic CT with intravenous contrast (A) Axial plane, without contrast. Portal vein with a hyperdense thrombus (circle), indicating that it is recent. (B) Atrophy of the right lobe of the liver (arrows) with no other signs of chronic liver disease - (B1) sagittal plane; (B2) axial plane. (C) Portal vein thrombosis (circle). Perivascular venous collaterals and at the level of the hepatic hilum (arrows); (C1) coronal plane; (C2) axial plane.

Abdominopelvic computed tomography (CT) with intravenous contrast showed an acute diffuse extensive venous thrombosis of the portal vein's axis with extension to the superior mesenteric and splenic veins, perivascular venous collaterals at the level of the hepatic hilum, and atrophy of the right lobe of the liver, equating possible antecedents of previous portal vein thrombosis. There were no other signs of liver disease or suggestive of acute infection (Figures 3A-3C).

The patient was admitted under enoxaparin anticoagulation. During the first five days of hospitalization, the abdominal pain resolved but remained febrile and with an increase in CRP and GGT in the analyses. Even though a clear focus of infection was not identified, the possibility of pylephlebitis was considered, given the suggestive findings. Blood cultures were collected, and empiric antibiotic therapy with piperacillin/tazobactam was initiated. Within 48 hours, the patient was apyretic, and the CRP was in regression. There was no evidence of malignancy in the performed study. Hepatitis B, hepatitis C, human immunodeficiency virus, syphilis, and cytomegalovirus serologies were negative. Autoimmunity testing was also negative, and there were no isolated microorganisms in blood cultures (Table 3).

**Table 3:** Laboratory investigation

Laboratory findings	Patient values	Reference range
Blood cultures	Negative	-
Hepatitis B	Non-reactive	-
Hepatitis C	Non-reactive	-
Human immunodeficiency virus 1 / 2	Non-reactive	-
Syphilis	Negative	-
Cytomegalovirus	Negative	-
Lupus anticoagulante	Negative	-
Cardiolipin antibodies	Negative	-
B2-glycoprotein-1 antibodies	Negative	-
Antinuclear antibodies (RNP70,A,C/Ro60,52/La/CentB/Scl-70/Jo-1/Sm)	Negative	-
Anti-Double-Stranded DNA Antibodies	Negative	-
Serum protein electrophoresis	Normal range	-
Complement C3 (mg/dL)	140,3	79-152
Complement C4 (mg/dL)	30,5	18-55
Immunoglobulin A (mg/dL)	289	70-400
Immunoglobulin G (mg/dL)	831	700-1600
Immunoglobulin M (mg/dL)	50	40-230
Sedimentation velocity (mm)	2	0-15

**Figure 4:**
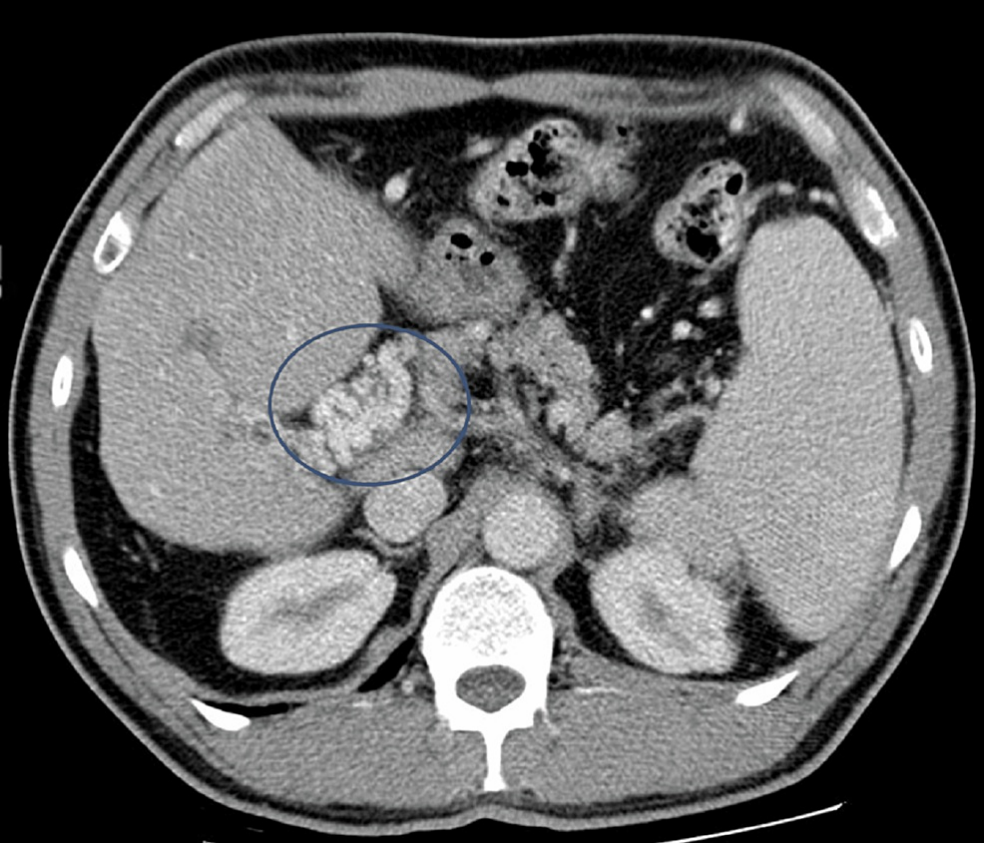
Abdominopelvic CT with intravenous contrast The total transformation into portal vein cavernoma (circle).

On the seventh day of intravenous antibiotic therapy, it was decided to change to an oral regimen with metronidazole plus levofloxacin. Low-molecular-weight heparin was altered to a vitamin k antagonist. The patient was discharged on the third day of oral antibiotic therapy with an indication to complete four weeks of treatment. Two weeks after discharge, he was reassessed in a consultation. He was asymptomatic, with sustained apyrexia and negative inflammatory parameters. Chest CT and upper and lower digestive endoscopy were performed and excluded malignancy. Abdominopelvic CT with intravenous contrast performed six months after the event showed a cavernous transformation (Figure 4). The patient remains anticoagulated and has had no other thrombotic events.

## Discussion

Hyperhomocysteinemia can be an inherited or acquired disorder and is a well-established risk factor for thrombosis [2,4,5]. Thermolabile enzyme MTHFR mutation, which plays a central role in regulating folate metabolism and homocysteine ​​synthesis, is the most common genetic alteration [2-6]. However, dietary deficiency of folic acid, vitamin B6, and vitamin B12; chronic diseases (such as high blood pressure or cholesterol, diabetes mellitus, obesity); kidney failure; hypothyroidism; medications (like as atorvastatin and fenofibrate) and other genetic abnormalities, can also increase homocysteine ​​levels [2,4-6]. A homocysteine level between 15 and 30 µmol/L is considered mildly elevated, between 30 and 60 µmol/L moderately high, and >60 µmol/L severely elevated [4,6].

Among MTHFR mutations, C677T and A1298C are the most investigated and lead to mild MTHFR enzyme deficiency [2,4,5]. MTHFR C677T mutation is the most common, which reduces enzyme activity by 65%. Another is MTHFR A1298C which leads to 60% of normal enzyme function [4,5]. Although an MTHFR enzyme reduced function can lead to elevated homocysteine levels, many people have normal homocysteine levels in countries where food is rich in folic acid [4].

The bond between MTHFR mutations, mild to moderate homocysteine elevations, and the risk of thrombosis has been central in current research [2,4,5]. In the past, studies associated hyperhomocysteinemia and MTHFR mutations with an increased risk of venous thrombosis. However, more recent studies admit that the association between mild hyperhomocysteinemia and VTE may be due to the confounders' presence [2,4-6]. In ClinVar, NM_005957.5(MTHFR):c.665C>T (p.Ala222Val), also known as MTHFR C677T, is associated with drug response, and NM_005957.5(MTHFR):c.1286A>C (p.Glu429Ala), also known as MTHFR A1298C, did not have any proven association. In case reports, these missense mutations are associated with homocystinuria due to methylene tetrahydrofolate reductase deficiency, folate-sensitive neural tube defects, and stroke. Still, the results have conflicting interpretations regarding pathogenicity [7,8]. Since clinical trials have failed to demonstrate that vitamin supplementation (folic acid, vitamin B6, and vitamin B12, either alone or in some combination) reduces levels of homocysteine ​​and recurrent VTE, assessment of homocysteine ​​levels or screening for MTHFR mutations in patients with venous thrombosis is not recommended [3,4,6].

Splanchnic-vein thrombosis has been reported as a complication of different pathologies, namely hypercoagulation disorders, abdominal trauma, intraperitoneal septic process, and cirrhosis, among others. It has variable consequences depending on its etiology and clinical presentation [9].

Pylephlebitis, also known as infectious suppurative thrombosis of the portal vein, can exacerbate intra-abdominal sepsis and has significant morbidity and mortality [5]. It starts with thrombophlebitis of small veins draining an infection site, making headway as septic portal thrombosis, and it can extend to hepatic, mesenteric, and splenic veins [5,10-13].

Acute colonic diverticulitis is the leading cause. Other possible sources are appendicitis, ileal diverticulitis, pancreatitis, cholangitis, cholecystitis, inflammatory bowel disease, bowel perforation, pelvic infections, a complication of diagnostic techniques, and surgical procedures [10,11]. A potential risk factor is a hypercoagulable state, either primary hematologic or other [11,13]. The precipitating factor is most often identified [9-12].

Clinical findings depend on the site, size, and severity of the primary inflammatory process. Fever, chills, malaise, upper abdominal pain, and tenderness are usually the initial features. Progression to sepsis associated with non-specific abdominal symptoms is typical [10-14]. Laboratory analyses are also wide-ranging. Traditional findings are anemia, leukocytosis, and abnormal liver function tests [9-11]. The diagnosis of pylephlebitis, often incidental, requires the demonstration of portal vein thrombosis (pylethrombosis) and is usually accompanied by bacteremia in a febrile patient [9,11,14]. The main diagnostic tests are ultrasound and CT scan [9,10,12].

The prevailing complications are the formation of liver abscesses and extension of thrombosis involving the superior mesenteric vein, the intrahepatic branches of the portal vein, and the splenic vein, which can lead to bowel ischemia and portal hypertension with venous collaterals [9-12]. The differential diagnosis includes intra-abdominal infections, namely cholecystitis, pancreatitis, intraperitoneal abscess, and hepatitis, among others [10].

Bacteremia associated with pylephlebitis is frequently polymicrobial. The most common bloodstream isolates are Bacteroides fragilis and Escherichia coli. Still, other organisms such as Aeromonas hydrophila, Streptococci, Proteus mirabilis, Klebsiella pneumonia, and Clostridium spp, among others, can also be isolated [10-12]. Pylephlebitis empirical antibiotic therapy “should include broad coverage for enteric facultative gram-negative bacilli, an agent active against anaerobes (especially Bacteroides fragilis), and range for aerobic Streptococcus species” [12]. Examples of recommended therapeutic regimens are combination therapy with metronidazole 500 mg every eight hours plus ceftriaxone 2 g daily or cefotaxime 2 g every six hours or ciprofloxacin 400 mg every 12 hours or levofloxacin 500 mg daily; monotherapy with a beta-lactam/beta-lactamase inhibitor, such as piperacillin-tazobactam 4.5 g every six hours or ampicillin-sulbactam 3 g every six hours; monotherapy with a carbapenem, such as imipenem 500 mg every six hours, meropenem 1 g every eight hours, or ertapenem 1 g daily [15]. Blood cultures should be obtained before the initiation of empirical therapy, with antibiotic choices adjusted when the Gram’s staining and culture results become available [15]. The perfect antibiotic duration is unknown [9-12]. However, given that hepatic abscesses are a common complication of pylephlebitis, and its development may not appear initially on CT scans, at least four weeks of antibiotic treatment is advised [9-12]. When hepatic abscesses are present, no less than six weeks of antibiotic therapy should be administrated, with or without drainage [12]. Surgery is performed electively to eradicate the primary inflammatory process when possible [9].

The purpose of anticoagulation in pylephlebitis has been dubious, primarily due to the absence of enough data [11,13]. One meta-analysis of one hundred case reports described a higher portal vein recanalization and lower mortality rate among anticoagulated patients. However, its conclusions were limited concerning potential confounders [10]. A retrospective study also showed that anticoagulation markedly improves the proportion of portal vein thrombosis resolution and substantially reduces the chronic symptomatic portal hypertension rate in pylephlebitis patients [13]. Beyond that, it showed a trend towards improved survival, without statistical significance, in patients on anticoagulation, apparently due to the contrast in the degree of acute illness and chronic disease in groups [13]. Thus, anticoagulation seems to be a vital component of the management of pylephlebitis and may be considered in selected cases such as patients with thrombosis progression on repeated imaging, continued fever, or bacteremia despite antibiotic therapy, thrombosis that extends beyond the portal vein into the mesenteric vein on initial assessment, and a hypercoagulable state underlying [12,13,16]. The perfect anticoagulation duration and agent remain unknown [13]. Traditionally, patients are anticoagulated with heparin derivatives in the acute phase, and subsequently, the switch to vitamin K antagonists is performed [12,13,16]. As of late, direct oral anticoagulants have also been used. Additional studies are needed to conclude its effectiveness [13]. It is reasonable to discontinue anticoagulation once the patient has improved clinically and imaging suggests that the thrombus is stable and not extending. These may occur within two to four weeks [12,13,16]. Often patients receive anticoagulation for at least three months, and recanalization has been documented in some cases, but the length of time required for this to occur is unknown [13,16]. In turn, the benefit of maintaining anticoagulation in patients with chronic portal venous thrombosis is less clear as the probability of recanalization diminishes in the chronic phase [13,16].

The presence of intra-abdominal infection, through the procoagulant effects of intestinal anaerobes and, perhaps, by the formation of neutrophil extracellular traps in local venules, causes peri- and intravascular inflammation in the splanchnic region, which is sufficient to provoke venous thrombosis [13]. So, seeking additional hematologic etiologies for VTE in pylephlebitis is commonly unnecessary [13]. A retrospective study showed that the only screenings for thrombophilia that tested positive at a remarkable frequency were those of dubious significance, such as MTHFR mutation and hyperhomocysteinemia. As they have not been consistently associated with VTE risk, they should not be included in thrombophilia assessments [13].

Currently, testing for hereditary thrombophilia remains controversial, data are limited, and there are no validated guidelines on this subject. Available data do not show significant differences in the rate of VTE recurrence between patients tested for hereditary thrombophilia compared with those who are not. So, the results of thrombophilia screening should rarely affect the treatment decision. The investigation of inherited and acquired thrombophilias may be considered in the following situations: VTE at a young age, often regarded as more youthful than 40-50 years old, in association with minor risk factors (such as minor surgery, immobilization, and oral contraceptive pills) or unprovoked VTE; first or subsequent VTE before 50 years and a strong family history of VTE; recurrent VTE; and VTE in an unusual site such as the central nervous system or splanchnic veins [3]. The concern regarding the cause and morbidity associated with splanchnic-vein or cerebral venous thrombosis often leads to testing for thrombophilia despite not being shown to play a role in the care [3]. Evaluation for paroxysmal nocturnal hemoglobinuria and myeloproliferative neoplasms must be taken into account in patients with unexplained splanchnic vein thrombosis because it can be its first manifestation [3,13].

In the case described, it was not possible to identify an acute intra-abdominal process, and there was no isolation of the etiological agent. Given the clinical and analytical improvement with treatment and the exclusion of other competing etiologies, such as malignancy and cirrhosis, the presumptive diagnosis of pylephlebitis was assumed. The patient evolved with signs of portal hypertension, venous collateralization, and cavernoma formation.

Despite the lack of formal indication for anticoagulation in patients with MTHFR A1298C mutation and mild hyperhomocysteinemia, it was decided to maintain oral anticoagulation with a vitamin K antagonist indefinitely as the patient already had two life-threatening thrombotic events (venous sinus thrombosis and splanchnic-vein thrombosis).

## Conclusions

VTE is a multifactorial process requiring physiologic, environmental, and genetic factors to precipitate thrombosis. Although hyperhomocysteinemia is an established risk factor for thrombosis, and MTHFR mutation is a recognized cause of hyperhomocysteinemia, the reduction of homocysteine ​​levels failed to reduce recurrent VTE. For that reason, screening for hyperhomocysteinemia and MTHFR mutation is not recommended. Pylephlebitis is a rare condition that can complicate intra-abdominal sepsis of any etiology and cause significant morbidity and mortality. Recanalization’s main factors are the early diagnosis of pylephlebitis and appropriate management of predisposing intra-abdominal infections. This last requires antibiotic therapy and anticoagulation in selected cases. The VTE approach must follow the current evidence with an integrative view of the patient and their comorbidities to offer the best treatment and result.
